# Detection of Single Molecules Illuminated by a Light-Emitting Diode

**DOI:** 10.3390/s110100905

**Published:** 2011-01-14

**Authors:** Ilja Gerhardt, Lijian Mai, Antía Lamas-Linares, Christian Kurtsiefer

**Affiliations:** 1 CQT, Centre for Quantum Technologies, 3 Science Drive 2, 117543, Singapore; E-Mails: maibb84@yahoo.com (L.M.); antia@quantumlah.org (A.L.L.); phyck@nus.edu.sg (C.K.); 2 Low Temperature Group, Chemistry Department, University of British Columbia, 2036 Main Mall, Vancouver, B.C., V6T 1Z1, Canada

**Keywords:** single molecules, fluorescence microscopy, light-emitting diode, LED, signal to noise ratio, single photon detection

## Abstract

Optical detection and spectroscopy of single molecules has become an indispensable tool in biological imaging and sensing. Its success is based on fluorescence of organic dye molecules under carefully engineered laser illumination. In this paper we demonstrate optical detection of single molecules on a wide-field microscope with an illumination based on a commercially available, green light-emitting diode. The results are directly compared with laser illumination in the same experimental configuration. The setup and the limiting factors, such as light transfer to the sample, spectral filtering and the resulting signal-to-noise ratio are discussed. A theoretical and an experimental approach to estimate these parameters are presented. The results can be adapted to other single emitter and illumination schemes.

## 1. Introduction

The invention of the laser in the 1960s was a key evolution in the path towards optical single molecule detection. Already in 1976 Hirschfeld performed an experiment, which was an important step towards this goal [1]. Using a laser to excite a fluorescently doped sample and detecting the spectrally filtered light on a photomultiplier, he was able to see the fluorescent fingerprint of a cluster of molecules. In the 1980s Moerner and Kador succeeded in the first optical detection of single pentacene molecules [2], but this new technique only became important for biology and sensing in the 1990s when the experiments were extended to work at room temperature. These experiments rely on efficient discrimination of the excitation laser light from the molecule’s red shifted fluorescence [3]. Since then, single molecule spectroscopy has become a valuable tool to overcome ensemble averaging over many emitters and to perform microscopy at a sub-diffraction limited scale [4,5]. In terms of sensitivity the detection of a single molecule of a certain compound represents the ultimate limit.

The above mentioned experiments were performed using laser illumination. The narrow linewidth and the coherent nature of the laser emission allow for spectral discrimination and easy focussing. Experiments performed using other light sources for single molecule research are rare [6–8]. In other fields, e.g., in white light imaging or fluorescence microscopy, the usage of non-laser sources is well established. At present, one of the most interesting light sources for microscopy is the light-emitting diode (LED), which underwent significant engineering efforts in the last two decades. High-power LEDs are commercially available, and could be thought of as compact and inexpensive sources for single molecule detection and sensing applications, or even for single photon generation. Their availability across the spectral range from 280 to 1,300 nm, their stable output, their electrical insensitivity, and long lifetime make them attractive alternatives to laser diodes for some applications.

In this paper we discuss the use of a commercially available LED as an excitation source for single molecule studies. Unlike presented before [7,8], we extend the experiments to the green part of the visible spectrum. The minimum irradiance to excite and detect single molecules is estimated by comparing the expected illumination levels to the nominal sensitivity of a camera and single photon detectors. A rigorous proof that a single molecule has been observed is only possible by detecting the characteristic anti-bunched photon statistics [9]. Prospects for the generation and detection of single photons based on LED illumination are discussed. Experimentally, we compare LEDs and laser excitation schemes side by side. It is shown that single molecules can be imaged with LED illumination and the influencing factors are presented. This extends the work of Kuo and coworkers, which mention present experimental findings on single molecule detection in the blue part of the spectrum [7].

## 2. Material and Methods

The sample for all further experiments and estimations consists of a doped thin crystalline film of *p*-terphenyl (Aldrich) which is spin coated on a microscopy coverslip [10]. As a dopant, the well characterized fluorescent molecule terrylene was chosen [11,12]. The concentration of terrylene molecules (PAH Research) is in the order of ≈10^−10^ to allow spatial separation. In an area of 10 × 10 *μ*m^2^ 1–20 molecules were observed. The coverslips were cleaned by organic solvents in an ultrasonic bath and afterwards transferred to a solution of 1 part sulfuric acid and 3 parts hydrogen peroxide (piraña solution) to clean organic residues. The coverslips continue to be submerged in the solution for storage until use and rinsed under water prior to spin coating sample preparation.

**Figure 1.**
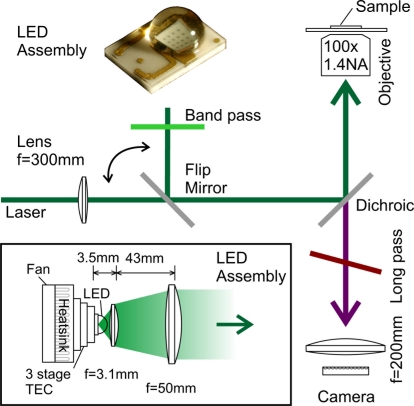
Experimental Setup, consisting of a confocal microscope (detection not shown) with wide-field configuration. A flip mirror allows to switch between laser and LED type illumination. Inset: Two lens LED-assembly, the LED is mounted with thermal grease directly onto a 3 stage thermo-electric cooler (TEC), which is attached to a fan-cooled heat sink.

All our single molecule studies were performed on a custom-made inverted microscope which can be configured for wide field or confocal imaging, and can use LED or laser illumination in either configuration (Figure 1). As a well characterized light source a frequency doubled Nd:YAG laser (532 nm) was coupled into the excitation path via a single mode optical fiber. To allow microscopy in wide-field mode, a f = 300 mm lens was placed in the optical path to focus the light into the backfocal plane of the microscope objective and to produce a Gaussian shaped illuminated area on the sample (full width, half maximum, FWHM ≈ 8 *μ*m). Illumination and detection were performed through a 100×, 1.4 NA objective (Olympus, UPlanSApo). The detection in wide-field mode was realized by a low-cost commercial astronomical camera (Watec, Wat-120N+, CCD: SONY ICX-419ALL). The image was captured with a 200 mm camera objective (AF Nikkor). A single pixel on the camera corresponds to the width of a standard deviation of the diffraction limited spot (*σ* ≈120 nm) on the sample, assuming the diffraction limited spot to show a FWHM of ≈300 nm. Thereby the pixel to spot size ratio is 1, as defined in [13], which is optimal for localizing single molecules. To record images, a video grabber card with a digitizing resolution of nominal 8 bit was used. The integration time of the camera could be set to a maximum value of 10.24 s.

For experiments using an LED, a flip mirror was introduced into the illumination path, such that either the laser or the LED light was passed to the sample, while the detection path remained unchanged. This allows for a comparison of the LED-based results with the well characterized laser illumination results. For laser illumination the detection was performed in confocal and wide-field configuration, whereas for LED illumination only wide-field images were recorded.

**Figure 2.**
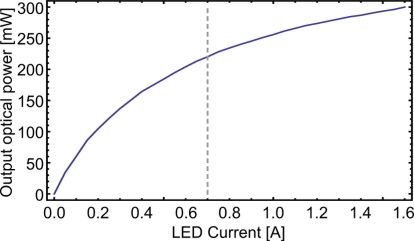
Measured light emission directly in front of light-emitting diode mounted in the diode assembly. The dashed line shows the nominal maximal current of 700 mA.

**Figure 3.**
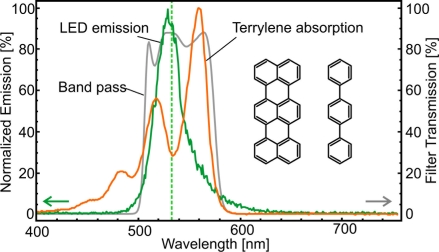
Absorption spectrum of terrylene and the emission spectrum of the unfiltered LED. The dashed line represents the wavelength of the frequency-doubled Nd:YAG laser. The irradiance of one to the other shows a by 40% larger value for the LED illumination. The larger spectral overlap allows a more efficient excitation. Inset: Terrylene (left) and the matrix molecule *p*-terphenyl (right).

The LED illumination source selection was based on the spectral overlap with terrylene absorption. Therefore commercially available green LEDs with a center wavelength around 535 nm were evaluated. The one with the highest irradiance was chosen for further experiments (Luminleds Luxeon Rebel, LXML-PM01-0080, InGaN). From a die surface of 1.6 × 1.6 mm^2^ an optical power of 240 mW at the nominal maximum current of 700 mA was detected on an optical power meter placed directly in front of the diode. The current could be increased to 1.6 A, resulting in P_out_ = 300 mW (≈180 lm, Figure 2), with proper cooling and by sacrificing the device lifetime. The light yield in this high power range is about 5%. On the die surface the emitted power corresponds to an exitance of 120 kW/m^2^. The wavelength shift over the entire current range was less than 5 nm from the peak wavelength of *λ* = 530 nm (see Figure 3). The spectral radiant exitance *M*_*λ*=530*nm*_ is 3,000 W/(m^2^ nm).

The thermal management of high power LEDs plays an important role for the device lifetime. Unlike in Reference [7,8] we utilize LEDs in the green region in the spectrum. Green LEDs have the lowest light yield of LEDs in the visible spectrum, whereas the earlier described [7,8] blue/UV LEDs have efficiencies up to 20% [14]. Our presented experiments require a much more careful designed cooling of the LED, but also might be extended to dyes which are presently more relevant in biological imaging, such as Cy3, Cy5 and other Alexa-Dyes in the red region of the spectrum. To allow an extended power supply to the LED, a three stage thermo-electric cooler (Ferrotec, 9530/119/045 B) was used to cool the LED base to temperatures below 0 °C. Under operating conditions, condensation was inhibited due to a higher thermal load of the LED.

Spectral filtering in the excitation path was performed with a 500–580 nm band pass filter, attached directly to the LED assembly (Photonik, Singapore, Figure 3). This filter was needed to suppress higher wavelength components of the LED emission, which were leaking through the detection filter. The integral LED transmission through the band pass filter was measured to be 70%.

**Figure 4.**
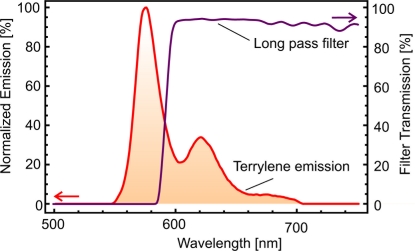
Emission spectrum of terrylene molecules. The optimal spectral filtering utilizes a similar slope to the emission spectrum of the molecule. In our experimental configuration a long-pass filter was slanted to match the excitation filter with a falling slope around 585 nm.

The detection path was equipped with a dichroic (50% Transmission/Reflection at 567 nm, Thorlabs DMLP 567) and a long pass filter with a nominal cut-off wavelength of 640 nm (Omega Optical 640AELP). The latter was slanted to match the cut-off wavelength of the exciter (see Figure 4 for the transmission of the slanted filter). The angle was tuned by observing the camera images for minimal background luminescence. The use of a dichroic mirror also reduces the transmission of excitation light towards the detector; a complementary pair of long pass and short pass filters should be sufficient, with the long pass filter at the detector and the short pass filter at the light source.

An optimal spectral filtering scheme is important to distinguish between excitation light and detected fluorescence, thus maximizing the signal to background ratio. Ideally, the filters would transfer all excitation light to the sample and simultaneously pass the entire resulting fluorescence to the detector. Simultaneously these filters have to block the excitation light scattered off the sample into the detection path. If a short pass/long pass filter set has complementary step transmission spectra around a cutoff wavelength *λ*_cut_, and the suppression of backscattered excitation light is taken care of, one can optimize the signal from a molecule by varying the cutoff wavelength for broadband sources like LEDs. For that, we combine the spectral power density of the source *l*(*λ*), the transmission *f*_exc_(*λ*) through all excitation filters, and the normalized absorption spectrum *a*(*λ*) of the molecule to an effective excitation flux *ϕ*_excitation_ by integration over all excitation wavelengths *λ_e_*:

(1) $$\varphi_{\text{excitation}}=\int_{\lambda_{\text{min}}}^{\lambda_{\text{cut}}}l(\lambda_{e})\;f_{\text{exc}}(\lambda_{e})a(\lambda_{e})\;{d\lambda }_{e}$$

It turns out that the normalized excitation flux for our LED is about 40% larger than for the laser at 532 nm due to a better spectral match.

If we assume that the fluorescence spectrum is independent of its excitation spectrum, which is the case for simple optical fluorescence configuration, the detected power is proportional to the product of this excitation flux. A detection path response *ϕ*_detection_ combines the emission spectrum *e*(*λ*) of the molecule, normalized to *e* = 1 in its maximum, the transmission *f*_det_(*λ*) through all detection filters, and the detector response *r*(*λ*) for all detection wavelengths *λ_d_*:

(2) $$\phi_{\text{detection}}=\int_{\lambda_{\text{cut}}}^{\lambda_{\text{max}}}e(\lambda_{d})\;f_{\text{det}}\;(\lambda_{d})r(\lambda_{d})\;d\lambda_{d}$$

For the combination of the LED and Terrylene emission/absorption spectra in our experiment (see Figures 3 and 4), a value of *λ*_cut_ = 565 nm maximizes the product *ϕ*_excitation_ · *ϕ*_detection_ and hence the detection signal.

One of the main advantages of coherent illumination is the constructive interference at the (laser) focus, reaching a maximum light intensity to effectively excite the molecule. Laser light can be focussed to a size approximately half the wavelength (*λ/*2), which is not possible for LED light. The limited phase-space density of incoherent light results in a larger illumination area and correspondingly lower irradiance. Usual LEDs exhibit a Lambertian spatial emission profile and are equipped with a solid immersion medium, consisting of either polycarbonate, acrylic glass, or, as in the used LED, silicon rubber for high power devices. This effectively increases light emission out of the die and concentrates light in the forward direction. Depending on the manufacturing accuracy of the die and the immersion medium the emission profile can vary significantly. To adapt the emission characteristics of the LED to the imaging geometry of the microscope objective, further collimation was necessary. For our experiments a high NA outcoupling lens (Geltech, C330TME-A, f = 3.1 mm, 0.68 NA) is placed very close to the LED. For this lens/LED combinations the solid immersion assembly was limiting the minimum focussing distance, thus limiting the effective outcoupling NA. The calculated radiance in one hemisphere is 20 kW/m^2^sr, taking the power of 300 mW, originating from an area of 1.6 × 1.6 mm^2^. With the aspheric lens in place and an assumed effective NA of 0.5, we expect a high outcoupling efficiency of 80%, if the emitted profile is purely Lambertian. The measured power behind the aspheric lens is 25% of the maximal measured power of the system right behind the die surface of the LED, suggesting a deviation of the Lambertian intensity profile and an effective lower NA. With these values, we calculate a radiance of 30 kW/m^2^sr. Assuming a focussing angle onto the sample of 3.9 sr (1.4 NA), this corresponds an maximum irradiance of 120 kW/m^2^ on the sample surface. The outcoupling aspheric lens was used in combination with a 50 mm achromatic lens to channel emitted light into the forward direction. The measured equivalent focal length of the system was 10 mm.

Due to the finite extension of the LED die and the incoherent light emission, the beam cannot be fully collimated. Initial attempts to filter the modes utilizing a single mode fiber were resulting in an outgoing power in the nW range and were not further pursued, such that wide-field illumination had to be used. To ensure an optimal transfer towards the sample and reduce clipping, the LED assembly was placed in close proximity to the microscopy setup. Initially the die was imaged to the backfocal plane of the microscope objective to reduce clipping in the optical path. This configuration resulted in a blurry die imaged on the sample and showed a lower intensity at the focal plane as independently measured with fluorescent beads. For later experiments the die was focused onto the coverslip such that its structure was visible on the wide-field camera if the spectral filters were removed.

## 3. Results and Discussions

### 3.1. Estimation of Minimum Irradiance for Detecting Single Molecules

As a first approach to estimate the minimum irradiance needed to detect single molecules which are illuminated with an LED we introduce a transfer expression. This relates the incident light to the detected outcome and compares it to the sensitivity and noise levels in the system. The interaction of light with a single emitter is determined by the effective absorption cross section of the emitter and the irradiance on this area, *i.e.*, the effective field strength at the location of the emitter and the excitation probability. The extinction cross section of a single molecule is in the order of *σ*_abs_ ≈ 1 × 10^−15^ cm^2^ [12,15]. By tightly focussing light down to a diffraction limited spot, diameter ≈300 nm, we have an effective overlap *σ*_eff_ of 1.5 × 10^−6^, *i.e.*, only one photon of 10^6^ is exciting the molecule, whereas the remaining light is non interacting or has an option to interact with the environment, leading to unwanted background. Depending on the fluorescence quantum yield Φ_fl_, and the molecules branching ratio *α*_branch_, only a fraction of absorbed photons leads to a red shifted emission which is later detectable. The integrated detection efficiency *η*_det_ for usual confocal microscopes is usually estimated to be 1–5%, and can be described by the following terms: The geometrical pickup *η*_geo_ is mainly determined by the numerical aperture of the microscope objective. In our experimental configuration with a 1.4 NA objective, we cover a half angle of 67°, corresponding to 30% of the entire emission in 4*π*. We detect light in the range of 590–700 nm, such that 45% of the spectral emission is transferred to the detector (*η*_spec_). All filters in the detection path were measured to have an integral transmission of better than 85% (*η*_filt_). Finally the quantum efficiency of our detector *η*_qe_ is in our detection range (590–700 nm) between 55% and 35%, whereas most of the light is emitted at longer wavelengths. This results in an effective detector quantum efficiency of 50%.

Assuming isotropic emission, the detected power *P*_det_ from the molecule reads as

(3) $$P_{\text{det}}=P_{\text{in}}\times \sigma_{\text{eff}}\times \Phi_{\text{fl}}\times \alpha_{\text{branch}}\times \eta_{\text{det}}$$

with

(4) $$\eta_{\text{det}}=\eta_{\text{geo}}\times \eta_{\text{spec}}\times \eta_{\text{filt}}\times \eta_{\text{qe}}$$

We assume the quantum yield of the molecule Φ_fl_ to be 0.7 [16] and a detection efficiency *η*_det_ of 5%. The branching ratio between red-shifted and resonantly scattered photons is assumed to be 1, because we excite into a higher vibrational level and consider the entire emission from the first electronic excited state. It follows that an incident rate of 1.5 × 10^10^ photons/s (corresponding to 80 kW/m^2^) onto the absorption cross section is needed to observe a flux of 800 detectable photons/s on the CCD camera. We now relate this rate to the detection sensitivity of the camera:

The camera’s minimal illumination is 2 × 10^−5^ lx, which corresponds to ≈40 photons per pixel and second in a spectral detection range of 590–700 nm. The measured noise-equivalent power with 10.24 s integration time was 36 photons per pixel. The molecule emits 15,000 photons per second, from which 800 per second are detectable, originating from a diffraction limited spot, which is imaged such that an average of 20% of its detectable emission is imaged on a single pixel. This delivers an illuminance of ≈8 × 10^−5^ lx or 160 photons per pixel and second, four times above the nominal minimum camera sensitivity.

The figure of merit in all experiments is the signal-to-noise ratio, which allows to differentiate between the actual signal and the background noise. The intrinsic noise sources such as photon shot noise, darkcount noise and background noise have to be included.

At the same irradiance levels as mentioned above we have a shot noise level of $\sqrt{800}$ photons/s. This value can be directly compared to the noise level of a single photon detector, such as an avalanche photo diode (APD). The given minimum illumination of the camera has to include the intrinsic signal to noise ratio and is already covered by our sensitivity assumptions above. When blocking the incident light on the camera and still ensuring the linearity over the whole detection range, we detect a background level of 13%, which would correspond to 270 dark counts per second on a single photon detector. The measured RMS noise of 2% corresponds to 36 photons per second. This value is approximately twice above the shot noise limited value of $\sqrt{270}$ photons. Interestingly the background level with LED illumination is 3–4 times higher than the camera background, due to imperfections of the spectral filtering. Since we are able to alternate between the two illumination options, we are able to exclude this effect to result solely from the background fluorescence of the sample. The intrinsic noise level of the camera does not allow us to associate an increase of the noise level due to the increased background and does not change significantly within the detection range.

### 3.2. Measurement of the Minimum Irradiance for Single Molecule Detection

To experimentally determine the minimum irradiance levels with LED excitation, we performed laser wide-field imaging and determined the minimum amount of irradiance, needed to observe single molecules on the camera. In these experiments, the integration time of the camera (10.24 s) and the gain were increased to the maximum and the laser power was subsequently reduced. At a laser irradiance threshold of 80 kW/m^2^ single molecules were still observable on a camera without any image postprocessing. The irradiance of the LED has nominally the same value, and given the 40% better spectral overlap this value should be sufficient for imaging.

**Figure 5.**
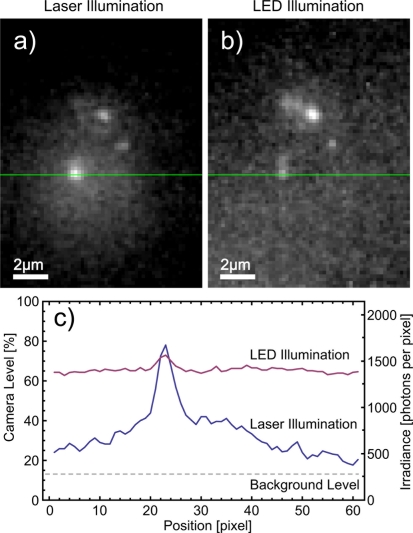
Direct comparison of two wide-field images acquired by laser **(a)** and LED illumination **(b)**. The gray levels of the images have been adapted to account for the increased background with the LED illumination. Still the slightly weaker signal to noise ratio is obvious. To have a direct comparison the camera levels are presented uncorrected in figure **(c)**. The background level of the camera is at 13% (blocking the excitation light) and should be subtracted for both illuminations. Light leakage through the filters increases the LED illumination background level to more than 60%.

Before observations are carried out with LED illumination, the spatial locations of individual molecules are determined using wide-field laser illumination with sufficiently high irradiance. At irradiance levels of about 80 kW/m^2^ fluorescence blinking cannot be observed due to the long integration time, whereas single step bleaching from frame to frame strongly indicates the detection of single molecule signals. After this, the excitation path was changed and alternating images were recorded with laser and LED illumination. An example is presented in Figure 5(a,b). The images are not processed, but only a fraction of the dynamic range is shown. We determined the laser wide-field illumination to be on a Gaussian area with FWHM of ≈8 *μ*m diameter. In the center the irradiance is about 80 kW/m^2^, and molecules are still observable at lower intensities in these images utilizing a smaller dynamical range. The effective pixel size is increased by the frame grabber card to ≈200 nm per pixel. In the measured microscope performance we achieve a slightly larger patterns as expected for diffraction limited microscopy. For LED illumination we achieve a FWHM of 330 nm, whereas the laser illumination leads to a FWHM of 430 nm. We attribute this deviation to a mechanical drift.

In Figure 5(c) no corrections have been made and the full camera range is shown. For the laser illumination we observe the wide-field illuminated area and a flat illumination background for LED illumination. The Gaussian intensity envelope in the laser excitation corresponds to the wide-field spot generated by the lens in the incident beam path.

For the laser and the LED illumination, we noticed a significant background contribution originating from residual fluorescence of the *p*-terphenyl. Here we underline that the direct comparison of the two illumination methods allows for a judgment on the sample quality and to differ this from spectral leaking through the excitation filters. In our experimental findings both parameters are not negligible and lead to a two times higher background intensity (see Figure 5(c)).

### 3.3. Detection of Single Molecule Signals

Although being at the lower limit of the excitation intensity we succeeded to detect single molecules with LED illumination in wide-field mode with a signal to noise ratio of 3.5. The corresponding signal to noise ratio generated by laser illumination was 19. The signal to background ratio is 0.12 and 1, respectively. To determine the presence of an object, the Rose’s criterion in imaging suggests a signal to noise ratio above 5 [17], which is not given in our configuration with LED illumination. However, with additional knowledge about the emitter, namely its nanometer size, resulting in a diffraction limited spot, we are able to determine single molecules against the noise floor of our detection scheme. As usual, wide-field detection allows us to use a localization algorithm to spatially localize the single emitters to a sub-diffraction accuracy. For samples with higher doping of fluorescent molecules the Rose’s criterion would indeed limit the probability to determine single molecule signals.

## 4. Outlook

The dual widefield/confocal configuration of this instrument is optimized for quick localization of single emitters and subsequent confocal imaging and measurement of individual molecules. We have shown that the current setup is able to reliably identify single molecules with both laser and LED illumination sources. To rigorously prove the presence of a single emitter one has to detect the single photon emission. Therefore the photon-photon autocorrelation function for zero time delay, g^(2)^(0), will then be below 0.5 [18]. However, it becomes clear from the LED based images presented above that this is not yet possible. The presence of other molecules or background light introduces additional photons, similar to having more emitters with a certain detection probability of *η*_i,det_ in the observation spot. The autocorrelation function for zero time delay can be expressed as $g^{\left(2\right)}(0)=1-1/(1+\sum_{i=1}^{n}\eta_{i,\text{det}}P_{i})$, where *n* is the number of additional emitters. The signal to background ratio (SBR) would be given by $\text{SBR}=1/(\sum_{i=1}^{n}\eta_{i,\text{det}}P_{i})$, such that the final equation reads *g*^(2)^(0) = 1/(1+SBR). When using higher laser intensities at a few hundred kW/m^2^ in a confocal configuration, we were able to quickly acquire autocorrelation signals with values below *g*^(2)^(0) = 0.2, thus proving the workability of the sample. In the case of LED illumination and a signal to background ratio of 0.12, it will not be possible to acquire an autocorrelation function below *g*^(2)^(0) ≈ 0.89. The noise and background floor need to be brought down by a factor of 9 for the dip in the *g*^(2)^(0) to fall below 0.5 as required. This problem might be circumvented by utilizing quartz coverslips to reduce fluorescence background and an optimized filter set with a cut-off wavelength at 565 nm. Another approach would be to spatially filter the emitted light more efficiently, such as in confocal imaging or to capture the light in a single mode fiber. In further studies we did not succeed in detecting single molecules with LED illumination in a *confocal* configuration. The spatial filtering by single mode fibers reduced the effective efficiency too drastically, and has also been reported by other groups [8].

To achieve a triggered single photon emission an optical pulse width below the molecules T_1_ time, *i.e.*, a few ns, should be used. Such LEDs are currently available on the market (e.g., Picoquant, PLS-series), but the irradiance is significantly smaller than the ones used in this paper. With the LED used here we were able to generate short pulses down to 100ns. The bond wires’ inductance might be the limiting factor in shortening the pulse for our device, but this was not further explored.

## 5. Conclusions

Performing single molecule spectroscopy with other illumination sources than lasers still remains a challenging task. The current efforts in engineering of semiconductor materials promise that light-emitting diodes will find their way into further single molecule studies in the future. The advantages of using LEDs are their compact design, their robustness, their intensity stability and their low cost, which make them a valuable tool to detect also small amount of fluorescent samples. Other particles like quantum dots might be even more successful candidates for LED excitation, because of their higher absorbtion in the blue region of the spectrum, opening the option for better spectral discrimination. It is an interesting option to illuminate vacancy centers in diamond, aiming for a triggered single photon source. Unfortunately usual vacancies in diamond (NV^−^) require a higher excitation irradiance and the detection relies on the red-shifted emission of the phonon wing. This makes the excitation and as well the discrimination between fluorescence and illumination photons even harder.
